# The Effect of Health Literacy on Men Seeking Medical Attention for Erectile Dysfunction

**DOI:** 10.7759/cureus.20424

**Published:** 2021-12-15

**Authors:** Arif Demirbas, Abdullah Gürel, Osman Gercek, Kutay Topal, Burhan Baylan

**Affiliations:** 1 Urology, Afyonkarahisar Health Sciences University, Afyonkarahisar, TUR; 2 Urology, Diskapi Yildirim Beyazit Training and Research Hospital, Ankara, TUR

**Keywords:** erectile dysfunction, health literacy, sexual dysfunction, socioeconomic level, andrology

## Abstract

Objective

In this study, we aimed to determine if there was any relationship between patients with erectile dysfunction (ED) who do not seek medical help at the hospitals and their health literacy (HL) levels.

Materials and methods

The study included 68 patients. Patients with a main complaint of ED were included in Group 1, and those with benign prostatic hyperplasia (BPH) or who requested an age-related check-up were included in Group 2. Questions 1-5 and 15 of the International Index of Erectile Function (IIEF)-15 scale evaluating erectile functions were collected as IIEF-6 for the purpose of this study. Severe ED was defined as having a score of 0-10, while moderate ED was defined as a score of 11-16. Both patients with severe and moderate ED were considered and included in the study. The European Health Literacy Scale (HLS-EU) and the Turkey Health Literacy Scale-32 (THLS-32) were used for the evaluation of HL.

Results

The HLS-EU person-specific mean index was found to be 37.22 ±5.29 in Group 1 and 30.46 ±6.32 in Group 2. The THLS-32 person-specific mean index was determined to be 37.68 ±5.41 in Group 1 and 30.94 ±6.13 in Group 2. In Group 1, 58.3% of patients were classified as having sufficient HL and 22.2% were classified as having excellent HL. In Group 2, 21.9% of patients were classified as having sufficient HL and 3.1% were classified as having excellent HL.

Conclusions

As societal income, education levels, and HL levels increase, ED will become more important to individuals, motivating them to seek timely medical attention, and thereby leading to earlier diagnoses of potential primary pathologies.

## Introduction

Erectile dysfunction (ED) is defined as a problem related to the onset or maintenance of an erection necessary for sufficient sexual satisfaction and performance, and various epidemiological studies have shown it to be a widespread issue. In previous studies involving various age groups, its prevalence has been determined to range from 19 to 52%, with the wide range in values attributed to differences in the definition of ED, calculation methods, patient groups selected, and socioeconomic and sociocultural differences among groups. Some sociocultural conditions are believed to result in a lower calculation of ED prevalence and incidence [1-5].

Recent studies have demonstrated the importance of having awareness regarding personal health protection and improvements during the course of a disease. With the development of health literacy (HL) scales to measure the level of this knowledge, researchers have attempted to understand the relationship between diseases and levels of HL. In individuals with insufficient HL, health protection, treatment compliance, and correct and timely diagnosis are all the more difficult. Although several tests have been designed for the measurement of HL, such as the Test of Functional Health Literacy in Adults (TOFHLA) and the Rapid Estimate of Adult Literacy in Medicine (REALM), the Health Literacy Survey-Europe (HLS-EU) has been reported to be a comprehensive and objective measurement questionnaire [6-9]. In 2016, following the validation of the HLS-EU by the Turkish Ministry of Health, the Turkish version of the European Health Literacy for Turkey was created. The same working group then developed the 32-item Turkey Health Literacy scale (THL-32) [10]. Based on the hypothesis that there is a direct relationship between sociocultural milieu and HL, we compared the HL levels of patients with ED who did not present to a healthcare institution for that reason and patients who presented because of ED. Thus, the aim of the study was to investigate if there was a relationship between patients with ED opting not to seek medical advice and their levels of HL.

## Materials and methods

The study was approved by the Afyonkarahisar Health Sciences University Clinical Research Ethics Committee (2011-KAEK-2 2020/137). The study included patients aged 50-75 years who presented at the urology clinics due to ED or symptoms of benign prostate hyperplasia (BPH) and voluntarily agreed to participate in the HL survey. Group 1 comprised patients with a complaint of ED, and Group 2 included patients who requested a check-up for BPH or because of age. The International Index of Erectile Function (IIEF), the Sexual Health Inventory for Men (SHIM), and the International Prostate Symptom Score (IPSS) for lower urinary tract symptoms (LUTS) forms were filled out by the participants and the results were recorded.

Patients with IPSS scores of >19 were excluded because severe LUTS may cause secondary ED. Questions 1-5 and 15 of the IIEF-15 scale evaluating erectile functions were collected as IIEF-6. Severe ED was defined as having a score from 0-10, while moderate ED was defined as a score of 11-16. Both patients with severe and moderate ED were considered and included in the study. To evaluate HL, the HLS-EU-Q47 and THL-32 scales were used. A record was also made of the education level of the patients (primary school, middle school, high school, university) and income status [≤2500 Turkish lira (TL), 2500-5000 TL, and >5000 TL].

The HLS-EU-Q47 total score was evaluated together with the general health literacy form (GEN-HL). Items 1-16 evaluated HL related to treatment and care (TC-HL), items 17-31 assessed HL on disease prevention (DP-HL), and items 32-47 examined HL on health promotion (HP-HL). A person-specific index was defined using the formula of arithmetic mean -1 x (50/3) [10]. The HL level was classified into four categories as in previous studies and based on validity and reliability studies. These four categories of HL were as follows: 0-25 points: insufficient HL, 26-33 points: problematic-borderline HL, 34-42 points: sufficient HL, and 43-50 points: excellent HL [10].

Statistical analysis

Data obtained in the study were analyzed statistically using the SPSS Statistics software version 23.0 (IBM, Armonk, NY). For the distribution of the data, the Shapiro-Wilk and Kolmogorov-Smirnov tests were used. These tests were used for variables with parametric distribution and the Mann-Whitney U test for non-parametric distribution. A p-value <0.05 was considered statistically significant.

## Results

A total of 68 patients were evaluated. Group 1 comprised 36 patients with a mean age of 63.27 ±6.70 years and Group 2 consisted of 32 patients with a mean age of 61.40 ±6.91 years (p=0.262) (Table 1). No statistically significant difference was found between the groups with respect to the IIEF, IIEF-6, SHIM, and IPSS scores used to measure erectile function and LUTS (p=0.769, p=0.398, p=0.183, and p=0.187, respectively) (Table 1).

**Table 1:** Descriptive and health literacy data of groups DP-HL: disease prevention health literacy; HLS-EU-Q47: Health Literacy Survey-Europe; HP-HL: health promotion health literacy; IIEF-15: International Index of Erectile Function; IIEF-6: International Index of Erectile Function, questions 1-5 and 15; IPSS: International Prostate Symptom Score; SD: standard deviation; SHIM: Sexual Health Inventory for Men; TC-HL: treatment and care health literacy; THL-32: Turkey Health Literacy scale

Variables	Group 1 (n=36), mean ±SD	Group 2 (n=32), mean ±SD	P-value
Age (years)	63.27 ±6.70	61.40 ±6.91	0.262
IIEF-15	20.55 ±7.22	20.09 ±5.43	0.769
IIEF-6	11.02 ±3.67	11.81 ±3.15	0.398
SHIM	9.33 ±3.16	10.31 ±2.97	0.183
IPSS	9.61 ±4.37	11.03 ±4.39	0.187
HLS-EU-Q47	37.22 ±5.29	30.46 ±6.32	<0.001
TC-HL	37.89 ±5.26	31.39 ±6.15	<0.001
DP-HL	37.10 ±5.29	29.93 ±6.51	<0.001
HP-HL	36.71 ±5.71	30.08 ±6.33	<0.001
THL-32	37.68 ±5.41	30.94 ±6.13	<0.001

The person-specific index of the HLS-EU-Q47 that is used to evaluate GEN-HL showed a mean of 37.22 ±5.29 for the patients in Group 1 who presented because of erection problems, and 30.46 ±6.32 in the Group 2 patients who presented with LUTS and were diagnosed with ED based on the results of their investigation forms (p<0.001). When both groups were evaluated with respect to the HLS-EU-Q47 subgroups, statistical significance was found in Group 1 for TC-HL, DP-HL, and HP-HL (p<0.001 for all) (Table 1). The person-specific index determined for the THL-32 had a mean of 37.68 ±5.41 in Group 1 and 30.94 ±6.13 in Group 2 (p<0.001) (Table 1).

When the GEN-HL of the groups was categorized, the distribution was as follows: insufficient HL in 2.8%, problematic-borderline HL in 16.7%, sufficient HL in 58.3%, and excellent HL in 22.2% of Group 1. The distribution of the patients in Group 2 was as follows: insufficient HL in 18.8%, problematic-borderline HL in 56.3%, sufficient HL in 21.9%, and excellent HL in 3.1% (p<0.001).

Statistically significant differences were found between the groups with respect to education levels and income. Patients who had completed only primary school comprised 13.9% in Group 1 and 37.5% in Group 2 (p=0.005). The level of monthly income was reported as ≤2500 TL by 13.9% of patients in Group 1 and 31.3% in Group 2 (p=0.004).

## Discussion

HL is defined as the capacity of a person to use healthcare services effectively and be able to access and appropriately interpret health information. Several studies have shown a correlation between the health data of a community and the HL level of individuals. The ability of an individual to understand and apply the information given by specialists and to know when and whom to consult for which problems are closely related to a good level of HL [11-13].

ED is defined as the inability to obtain an erection sufficient for desired sexual performance [14]. As previous studies have reported a wide range of ED prevalence, it can be understood that it is difficult to determine the actual values related to it. In previous research, sociocultural levels have been a determinant of increased incidence and prevalence. In a longitudinal study of 1156 patients with a median follow-up period of 8.8 years, Johannes et al. determined that the incidence of ED decreased when education and socioeconomic levels were high [15].

Although published studies have researched the relationship between HL and several pathological conditions, especially chronic diseases, no information could be found in the literature with respect to the evaluation of the relationship between ED and HL. The results of this study showed a strong relationship between ED and HL, just as for pathologies in previous studies. In this study, patients who presented directly with ED had generally high GEN-HL levels: 58.3% had sufficient GEN-HL, while 22.2% had excellent GEN-HL. However, patients who presented with LUTS and were later determined to have ED had lower GEN-HL levels, with 21.9% of these patients having sufficient GEN-HL and only 3.1% having excellent GEN-HL. This relationship was also valid for the subgroups of HLS-EU, the TC-HL, DP-HL, HP-HL, and the THL-32 developed by the Turkish Ministry of Health, where it was seen that the level of HL was higher in the patients who presented with ED. When education and income levels were compared across groups, patients with a presenting complaint of ED demonstrated higher levels of both.

The findings of this study showed that HL levels of patients consulting a urology specialist because of ED were higher than those of patients who have ED but present with a different complaint. This suggests that as HL levels increase in tandem with community income and educational levels, ED, which is a symptom of conditions such as diabetes and coronary artery disease, will become more important to individuals, leading them to seek medical evaluation earlier and ultimately resulting in an earlier diagnosis of potential primary pathologies.

This study has a few limitations. These include the self-reported nature of the data collected in the forms, the small sample size, and the single-center study design. Hence, further studies with larger sample sizes are needed to validate our findings.

## Conclusions

As in other diseases, increased HL levels will have a positive impact on ED diagnosis. As HL levels increase, ED, which is a symptom of conditions such as diabetes and coronary artery disease, will become more important to individuals. This may lead them to seek medical attention earlier and hence result in a more timely diagnosis of primary pathologies. We recommend further studies with larger sample sizes to validate these findings.
